# Improving the operational efficiency of Phase 2 and 3 trials

**DOI:** 10.1186/s13063-016-1465-3

**Published:** 2016-07-20

**Authors:** Jitendra Ganju

**Affiliations:** Global Blood Therapeutics, South San Francisco, CA 94080 USA

**Keywords:** Blinded clinical trials, Risk reduction, Unblinding

## Abstract

The period toward the end of patients’ participation in late stage blinded clinical trials is highly resource intensive for the sponsor. Consider first a Phase 3 trial. If the trial is a success, the sponsor has to implement the next steps, which might be filing for approval of the drug with the US Food and Drug Administration (FDA). To shorten the time interval between trial completion and submission of the package to the FDA, sponsors front-load as much work as is possible at risk. The approach is efficient if the trial succeeds but is inefficient if it fails. For a failed trial, the sponsor is unlikely to proceed with the plan that assumed success. Phase 2 trials are also at risk of being inefficient. Many activities, such as planning for drug interaction studies, thorough QT studies, or site selection for Phase 3 trials, are set in motion prior to completion of the Phase 2 trial. The work going on in parallel is wasted if the trial fails. The proposal to improve the efficiency is to let an independent entity provide the sponsor critical information at an earlier time necessary to reevaluate activities ongoing in parallel and external to the trial.

## Background

The timelines for achieving certain milestones following the completion of a Phase 2 or 3 randomized, double-blind clinical trial are very aggressive, as the stakes are high. The practice in industry is to compress the time between the release of results and the next step, assuming a successful trial. The next step for a Phase 3 trial might be submitting a New Drug Application (NDA) to a health agency such as the US Food and Drug Administration (FDA). Or it might mean concurrently planning for trials in a different age range or in related indications. For a Phase 2 trial, the next step might be any combination of the following activities: identifying sites for a global Phase 3 trial, planning and conducting a thorough QT study and drug interaction studies, or preparing to meet with health authorities (known as an End of Phase 2 [EOP2] meeting) to clarify aspects of the sponsor’s drug development plan.

Compression of timelines is achieved by front-loading the tasks while the Phase 2 or 3 trial is still ongoing. The effort is undertaken at risk, and is of necessity highly labor intensive. Front-loading is a highly coordinated activity. Various functions within the sponsor’s organization — project management, commercial operations, manufacturing, drug safety, biostatistics, clinical, clinical operations, data management, statistical programming, regulatory, medical writing — all work in lockstep, while the trial is still blinded, to complete their tasks in a timely manner.

Front-loading pays off for a successful trial but it is inefficient if the trial fails. Lack of attention to other development areas exacerbates the inefficiency. Some examples of at-risk work that may become unnecessary if the trial fails include preparation of documents for submission to regulatory agencies or initiating steps to plan additional trials. The purpose of this article is to propose a way to improve the process by providing the sponsor limited but important information at an earlier time to reduce the risk of wasting resources.

To understand the proposal, it is helpful to outline the sequence of steps after the last patient is enrolled. After completion of enrollment, patients are followed until their last visit. Before and after completion of enrollment, data are collected, entered in the database, and “cleaned” (i.e., checked for errors and logical consistency). Different data elements follow their own cadence from data capture to availability in the database. Some data elements are available sooner than others; however, the standard practice requires all data to be fully available in the database prior to its lock. The different data elements fall into the following broad categories:

- Safety data, which come from different sources including reports of adverse events by the investigators, results from central and local laboratories, and electrocardiograms
- Efficacy data on the primary and secondary endpoints, which also come from different sources. Examples include: patient-reported outcomes (e.g., disability index, quality of life questionnaires), assessment made by the patient’s site staff (e.g., signs and symptoms), and adjudicated endpoints (e.g., stroke or myocardial infarction)
- Other data such as medical history, demographics, and concomitant medications

After the database is locked, the study is unblinded. By unblinding after database lock, we mean the release of unblinded study results. The time interval between availability of almost all or all data and unblinding varies by therapeutic area and other factors, ranging from one month to several months.

The proposal to improve the operational efficiency will benefit trials with a large time window between data availability and unblinding. An example of such a trial is a global, endpoint-adjudicated, event-driven trial. Event-driven trials have a common closure date; that is, the final visit is approximately the same for all trial patients in calendar time. The final visit occurs after accrual of the requisite number of events and achieving of the protocol-stipulated minimum trial size and minimum duration of follow-up. Projecting when the final visit will occur helps to determine the final visit date. Estimating the timing of the final event and arranging for (approximately) the same final visit date across all global sites is complex and takes time to execute. Further, the endpoint information provided by non-English speaking sites has to be translated into English and then sent to an endpoints committee who meet at scheduled times to determine the absence or occurrence of an event. These steps usually mean that the time window between availability of almost all the data on the endpoint and database lock is large.

What information to release to the sponsor, the timing, and the benefit are explained next.

## Main text

### The information given the sponsor

What information must the sponsor receive? The recommendation is a restricted version of the result on the primary efficacy endpoint, such as whether a certain condition is met. The condition proposed is if the *p* value for the primary endpoint, denoted *p*, exceeds a certain prespecified threshold *p*_1_. The actual value of *p* is not provided. All the sponsor is told is whether *p* > *p*_1_. We call this the “high level analysis.” The method of analysis to calculate *p* is the same as the one described in the statistical analysis plan.

The choice of *p*_1_ is important. The idea is to select a value such that if *p* > *p*_1_, it is understood that the trial has failed with respect to the primary endpoint. *p*_1_ should be large enough that *p* ≤ *p*_1_ does not suggest success but not so large that the probability *p* > *p*_1_ is low under the null. A value of *p*_1_ in the range of 0.20—0.30 seems reasonable. For example, suppose we choose *p*_1_ = 0.25. *p* > 0.25 means a negative result on the primary endpoint, whereas *p* ≤ 0.25 does not imply success.

An independent entity external to the sponsor can undertake the task of performing the analysis and provide the information to the sponsor. Most Phase 3 and many Phase 2 trials have data monitoring committees (DMCs) to monitor the safety and efficacy. If agreeable to the DMC, they can relay the necessary information to the sponsor. To maintain trial integrity, the burden is on the sponsor to document all steps, including who within the sponsor’s organization will receive the result, and to make the process transparent. A choice of *p*_1_ that is small (e.g., 0.10) is not advisable because of the risk it poses to the integrity of the trial. A result that *p* ≤ *p*_1_ = 0.10 may be interpreted as a successful trial even though the results on secondary endpoints (some perhaps as important as the primary endpoint) and the risk-benefit assessment are still pending. To be informed that the chance of success has increased because *p* ≤ *p*_1_, when the sponsor is already working at risk assuming a successful trial, is not necessary. It raises suspicion because the sponsor is given information that ought not to matter.

### Timing of analysis including release of result to sponsor

The sponsor should receive the result (i.e., whether *p* > *p*_1_) late in the course of the blinded trial. Late means that no changes can be made to the trial itself, but knowing the result allows for affecting parallel activities external to the trial. This suggests a time close to the availability of all data for analysis of the primary endpoint. Conducting the analysis when almost all the data are available will ensure that *p* and the *p* value calculated after official unblinding are similar. Changes to the statistical analysis plan after the release of the high level result should not involve the primary endpoint and any closely related secondary endpoints (e.g., endpoints that are components of a composite primary endpoint). No alpha adjustment is necessary because there is no change to the conduct or to the analysis of the ongoing trial. The proposal is not to be mistaken for a futility analysis, which permits the early stopping of a trial if the interim results are not promising [1].

To make concrete how the timing of the analysis might work, we continue with the global, event-driven, endpoint-adjudicated trial. Suppose the trial design is such that 500 events and a minimum follow-up of one year are required. Assume that as the trial progresses it becomes apparent that the 500th event will occur prior to the completion of the minimum follow-up. A projection made around the time of the occurrence of the 400th adjudicated event suggests that the 500th investigator-reported event (which is to be adjudicated) will occur 4 months before the minimum follow-up is complete. Suppose further that the concordance between investigator-reported and endpoint committee events is high [2]. Additional events may occur between the occurrence of the adjudicated 500th event and minimum follow-up which would be adjudicated and included in the primary (official) analysis but not included in the high level analysis.

Rather than wait for adjudication and the minimum follow-up to be complete for all patients, suppose the high level analysis is conducted soon after the occurrence of the investigator-reported 500th event. The analysis would include all adjudicated events, and investigator-reported events would be included only for events that are pending adjudication. While the focus is on event-driven trials, the proposal may hold for visit-driven trials as well if the time between the last patient visit and database lock is long.

Two situations warrant additional consideration regarding timing. For the first consideration, the analysis will need to occur later than when primary endpoint data are available. If the patient follow-up for the primary endpoint is less than that for other endpoints, the proposed analysis must occur closer to the time of the final visit to prevent any modifications to the trial. For the second consideration it may occur earlier. A few stragglers can drag out the time to trial completion either because of how late they enrolled or because their actual final visit is known in advance to occur much after the scheduled final visit. In such a case, the analysis can occur sooner by excluding their final visit data, and it assumes that the consequence of data omission is expected to be minimal.

### Benefit

Suppose the sponsor is told that *p* > *p*_1_. Then at-risk tasks can stop or move at a slower pace. Sponsors will likely reallocate resources and other development areas will automatically receive more attention. In all other respects, the trial continues through completion. If instead the sponsor is told that *p* ≤ *p*_1_, the at-risk tasks continue as per plan.

One possible objection to the proposal may be: Why should anyone other than the sponsor care? After all, the gain is merely related to operational efficiency. No ethical or public health matters are at stake. The response to that is twofold: (1) What is proposed is an option for sponsors to consider. Currently, tradition mandates how to unblind all late stage trials. Convention should guide, not dictate, how trials should be run. For some sponsor organizations, particularly ones with limited financial resources, the gain can be substantial. It could be argued the gain in efficiency could indirectly benefit public health since the limited resources would be directed to other more promising development programs. (2) A wider recognition of the cost that the current system imposes itself has value.

## Conclusion

Releasing the high level result earlier is not appropriate for all trials: (1) A trial for which all data become available soon after the final visit is unlikely to be a good candidate, and neither is (2) a trial in which the sponsor will continue its at-risk tasks as planned even if *p* > *p*_1_ (because, say, of the importance of the secondary endpoints). One risk with the proposal is the possibility of decline in the quality of data collected toward the end if the sponsor is told that *p* > *p*_1_. While this is possible, it seems unlikely, as the processes to see the trial through to completion will have already been in place since the start of the trial. Another risk might be if the information that *p* > *p*_1_ is leaked and is in the public domain before official unblinding. This could complicate matters for the sponsor if the external community reacts to the result before the sponsor has had a chance to understand the totality of the trial data. To avoid this risk, the sponsor has to be committed to a disciplined approach and judiciously choose the timing for release of such information.

A press release may follow on the heels of a completed trial. Whether this is to occur soon after limited early unblinding will need to be thought through in advance.

## Abbreviations

DMC, data monitoring committee; EOP2, End of Phase 2; FDA, Food and Drug Administration; QT interval, measure of time between the start of the Q wave and end of the T wave in the heart
